# Improving Electrical Stimulation Effectiveness and Versatility for Non-Invasive Transdermal Monitoring Applications via an Innovative Mixed-Signal Electronic Interface

**DOI:** 10.3390/s24237626

**Published:** 2024-11-28

**Authors:** Alessandro Zompanti, Davide Ciarrocchi, Simone Grasso, Riccardo Olivieri, Giuseppe Ferri, Marco Santonico, Giorgio Pennazza

**Affiliations:** 1 Research Unit of Electronics for Sensor Systems, Department of Engineering, University Campus Bio-Medico di Roma, 00128 Rome, Italy; davide.ciarrocchi@unicampus.it (D.C.); g.pennazza@unicampus.it (G.P.); 2 Research Unit of Electronics for Sensor Systems, Department of Science and Technology for Sustainable Development and One Health, University Campus Bio-Medico di Roma, 00128 Rome, Italy; s.grasso@unicampus.it (S.G.); m.santonico@unicampus.it (M.S.); 3 Department of Industrial and Information Engineering and Economics (DIIIE), University of L’Aquila, 67100 L’Aquila, Italy; riccardo.olivieri1@student.univaq.it (R.O.); giuseppe.ferri@univaq.it (G.F.)

**Keywords:** current stimulation, electrical stimulation, drug delivery, functional electrical stimulation, mixed-signal interface, reverse iontophoresis, transdermal monitoring

## Abstract

Electrical stimulation can be used in several applications such as fatigue reduction, muscle rehabilitation, neurorehabilitation, neuro-prosthesis and pain relief. Moreover, electrical stimulation can be used for drug delivery applications or body fluids extraction (e.g., sweat and interstitial fluid) to successively monitor several parameters, such as glucose, lactate, etc. All these applications are performed using electrical stimulator devices capable of applying constant voltage pulses or constant current pulses via electrodes to human tissues. Usually, constant current stimulators are most widely used because of their safety, stability, and repeatability. Thus, the aim of this work was to design, realize and test a mixed-signal electronic interface capable of producing current pulses with custom amplitude, duration, frequency, polarity and symmetry with extended voltage compliance. To achieve this result, we developed a high-voltage current stimulator suitable for iontophoresis applications. Current stimuli can be applied setting the intensity, frequency and duty cycle of the stimulation patterns through a µC. A custom electronic interface was designed to allow the control of the injected current in real time and to prevent electrical injuries to the patient by avoiding potential unwanted short circuits. Moreover, the system was tested in a simulated environment demonstrating its effectiveness and applicability for transdermal monitoring applications. The obtained results show that the device is able to apply monophasic and biphasic pulses, ranging from 0.1 to 10 mA, with a maximum error of about 10% at the minimum intensity; in addition, current stimuli can be applied up to a maximum frequency of 100 kHz with a voltage compliance of 120 V.

## 1. Introduction

Electrical stimulation can be performed by injecting small amounts of current through nerves, muscles, and skin by applying electrical pulses. Electrical stimulator devices can apply constant voltage pulses or constant current pulses through electrodes. Impedance between electrodes can be affected over time by several factors, such as sweat, temperature, etc., and may change between different patients, so applying the same controlled voltage pulses could not be sufficient to inject the same amount of current into the tissues and to produce the same desired stimulation. Thus, constant current stimulators are most widely used because of their stability and repeatability. On the other hand, the injection of even a small current (1–10 mA) through a high impedance (10–50 kΩ) will require high voltages.

Electrical stimulation can be used in several clinical applications for therapeutic purposes. The most common electrical stimulation techniques applied in fatigue reduction, muscle rehabilitation, neuro-rehabilitation, neuro-prosthesis and pain relief are Electrical Muscle Stimulation (EMS), Transcutaneous Electrical Nerve Stimulation (TENS), Functional Electrical Stimulation (FES) and Neuromuscular Electrical Stimulation (NMES) [1,2,3,4,5,6,7,8,9].

Moreover, electrical stimulation can be used for drug delivery applications, using iontophoresis [10], or to monitor blood parameters (e.g., glucose, lactate, etc.) using reverse iontophoresis [11,12,13,14].

Iontophoresis is a non-invasive technique used to increase the transdermal penetration of substances, especially drugs, through the skin layers [15,16]. Iontophoresis is a process of transdermal drug delivery that uses a voltage gradient on the skin. The transport of molecules across the stratum corneum is facilitated by electrophoresis and electroosmosis, which are phenomena that constitute active transport of matter due to an applied electric current. Thus, iontophoresis can be achieved by applying small electric current stimuli through electrodes applied on the skin.

Reverse iontophoresis is a non-invasive technique used in non-invasive monitoring [17], while iontophoresis is used to enhance the transport of drugs across the skin, by applying small electric current signals, reverse iontophoresis is used to extract substances from the interstitial fluid (ISF) to the skin surface for health monitoring purposes, by applying a small electric current as well. ISF is a body fluid and contains several important analytes, such as glucose, lactate, and urea [18]. Those analytes are related to those present in blood [19] and are really useful to monitor physiological parameters. Moreover, the analytes contained in ISF can be sampled in a non-invasive or minimally invasive way.

Non-invasive sampling techniques offer several benefits, including the ability to obtain more information through frequent sampling, better compliance due to decreased pain and discomfort and reduced risk of infection. On the other hand, the quantity of analyte obtained using this technique is small and dilution factors are high, so analytical methods or devices with higher performances, such as sensitivity, LOD, etc., must be used. This means that clinical chemistry laboratory equipment could be necessary, which would cause a limitation in the use of wearable devices and a lengthening of sampling time and measurement intervals. For some applications, such as drug monitoring, ones this could be acceptable, but not for continuous monitoring applications, such as glucose monitoring. Thus, it is crucial to maximize the capabilities of the reverse iontophoresis and simultaneously the performances of measuring wearable devices to allow at-home applications in real-life scenarios.

To date, the only device exploiting reverse iontophoresis to have been commercialized is the GlucoWatch G2 Biographer (Cygnus Inc., Redwood City, CA, USA). This device was commercialized in 2001, but was discontinued in 2007 due to device problems. The device was able to monitor blood glucose levels in a non-invasive way. This device extracted glucose through intact skin via reverse iontophoresis where it was detected by an amperometric biosensor [20]. One of the major causes of the commercial failure of GlucoWatch was skin irritation due to extended stimulation. This kind of issue could be minimized, improving the conductivity between the skin and the electrodes and using proper current pulses. Thus, it is crucial to develop an electronic interface capable of applying tunable current pulses.

Moreover, some research devices can be found in literature, demonstrating a certain interest of the scientific community in this technology. Ching et al. [21,22,23] developed low-cost and programmable devices capable of delivering current stimuli for biomedical applications. Anisimov et al. [24] developed a portable device for Iontophoresis, and Bok et al. [25] developed a portable ionic device for efficient transdermal drug delivery using electric fields.

In recent years, the field of flexible electronics and materials science has witnessed significant advancements. These developments have led to a qualitative improvement in the application of reverse iontophoresis [26]. In the last five years, some applications of reverse iontophoresis have been explored [27]: Cheng et al. [28] developed a non-invasive biosensor for monitoring glucose in ISF exploiting a solid microneedle array to penetrate the external skin layers; Xu et al. [29] developed an electrochemical biosensor based on PEDOT:PSS conductive hydrogel for the non-invasive and continuous monitoring of glucose; Xi et al. [30] developed a closed-loop system capable of tracking blood glucose in a mini-invasively way using microneedles; Pu et al. [31] developed a flexible device to monitor blood glucose using a thermal activation method to improve the efficiency of reverse iontophoresis extraction; Giri et al. [32] investigated ex vivo and in vivo transdermal extraction of levetiracetam across the pig ear skin via reverse iontophoresis; Yang et al. [33] extracted a cancer biomarker through reverse iontophoresis combined with microneedles; Ventura et al. [34] achieved the extraction and monitoring of cortisol via reverse iontophoresis.

Thus, as demonstrated in the literature, ISF extraction and analysis could be effective to monitor the health status of a patient with an innovative, minimally invasive approach.

Within this context, the aim of this work is the design, development and testing of a novel mixed-signal electronic interface able to produce current pulses of a custom shape (duration, frequency, polarity) and to test the system in a simulated environment to prove its effectiveness and applicability for transdermal monitoring applications.

## 2. Materials and Methods

**Figure 1:**
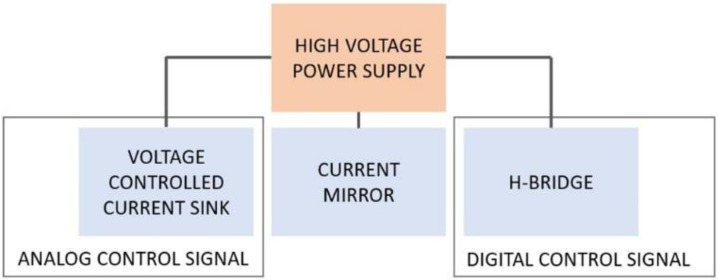
Block diagram of the developed electronic interface.

The developed device is composed of four main functional blocks, as shown in Figure 1:

- High voltage power supply: this block is needed to supply high voltages if stimulating high impedance loads;
- Voltage-controlled current sink: this circuital block is used to inject the desired amount of current into the stimulated tissue, regardless of its impedance (within a certain range); a current sink topology was chosen over a current source topology in order to allow the use of low voltages as the control signal, produced directly by a DAC without the need of a further condition (e.g., amplification stages);
- Current mirror: the current mirror block is needed to apply to the stimulated tissue the current produced by the current sink;
- H-bridge: the h-bridge block is used to apply biphasic stimuli to the tissue without the need of a dual power supply.

The high voltage power supply is based on the R05-100B (RECOM Power GmbH, Austria), a current mode DC/DC switching converter operating from a 4.5 V to 6 V input value. The output voltage is equal to 120 V with an input voltage of 5 V, and the maximum output current is equal to 25 mA.

**Figure 2:**
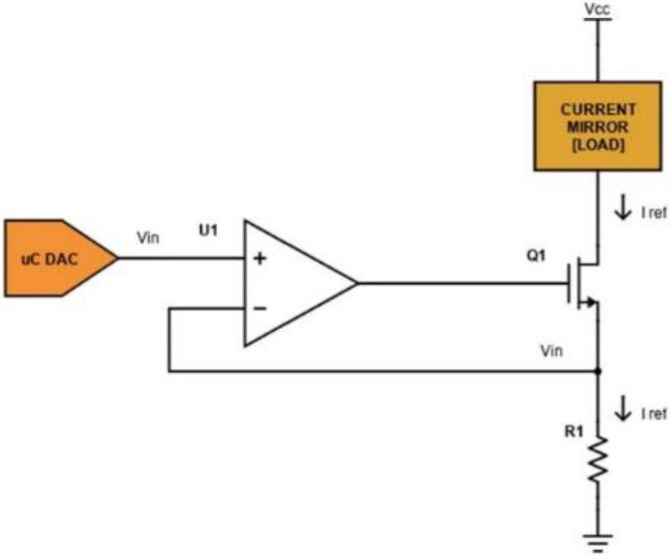
Schematic of the voltage-controlled current sink circuit.

The voltage-controlled current sink block is implemented as shown in Figure 2. The desired current is set by providing a voltage, *V_in_*, on the non-inverting input pin of the op-amp U1 (an ADA4505, Analog Devices), through the internal DAC of a µC (SAM3X8E ARM Cortex-M3, Atmel, San Jose, CA, USA).

The current *I_REF_* flowing on the load is given using the equation:

(1) $$I_{REF}=\frac{V_{in}}{R_{1}}$$

**Figure 3:**
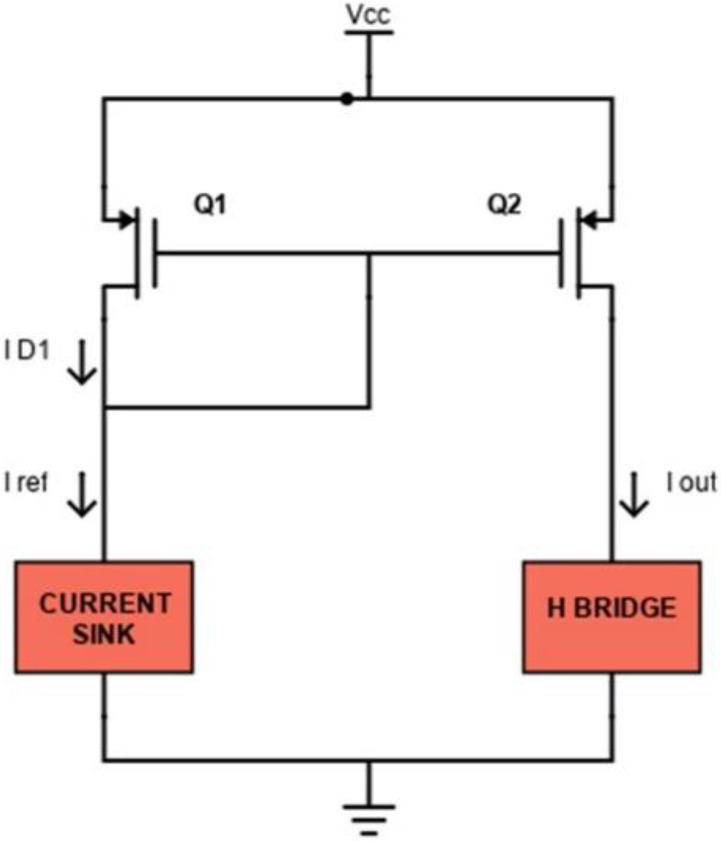
Schematic of the current mirror circuit.

In fact, as a current flows through the load, a voltage is obtained on *R*_1_. The op-amp uses the sensed voltage as feedback and drives its output until the sensed voltage is equal to the DAC output *V_in_*. In this case, transistor *Q*_1_ (we have used the commercial device FDN86246, Onsemi, Scottsdale, AZ, USA) is working in the linear region as a voltage-controlled resistance. The used DAC supports 12-bit resolution with a 3.3 V power supply: if *R*_1_ is equal to 100 Ω, the sink current theoretically ranges from 0 to 33 mA with a minimum step of 8 µA (from Equation (1)). A voltage *V_cc_* equal to 120 V is provided as a high voltage power supply stage. The current mirror block is implemented as shown in Figure 3.

The P-MOS transistor *Q*_1_ (ZVP1320FTA, Diodes Incorporated, Plano, TX, USA) works in saturation mode because the drain is shorted to its gate. In this region, the thickness of the channel at the drain decreases until it nullifies, and it is said that the channel is in pinch-off, for Δ*L* tending to zero. The channel length decreases by an amount Δ*L*. As *V_ds_* increases, Δ*L* increases, so in the saturation region, the slope of the curve also depends on *V_ds_* through the channel modulation factor λ. It is possible to approximate λ as a parameter inversely proportional to the channel length as:

(2) $$l=\sqrt{\frac{\varepsilon }{qL^{2}N_{A}}}\approx \frac{10^{7}}{L\sqrt{N_{A}}}\approx 0.1\;V^{-1}$$

for typical *L* values in the simulation range.

In this case, the drain current *I_D_*_1_ is equal to *I_REF_* given by:

(3) $$I_{D1}=\frac{1}{2}{\left({µ}_{p}C_{ox}\frac{W}{L}\right)}_{Q1}{\left(V_{GS}-V_{TH}\right)}^{2}\;(1+lV_{ds})$$

If also *Q*_2_ (ZVP1320FTA, Diodes) is operating in saturation, its current is equal to:

(4) $$I_{D2}=I_{OUT}=\frac{1}{2}{\left({µ}_{p}C_{ox}\frac{W}{L}\right)}_{Q2}{\left(V_{GS}-V_{TH}\right)}^{2}(1+lV_{ds})$$

so the relation between *I_OUT_* and *I_REF_* can be given by the following expression:

(5) $$\frac{I_{OUT}}{I_{REF}}=\frac{I_{D2}}{I_{D1}}=\frac{{\frac{1}{2}\left({µ}_{p}C_{ox}\frac{W}{L}\right)}_{Q2}{\left(V_{{GS}_{2}}-V_{TH}\right)}^{2}{\left(1+lV_{ds}\right)}_{Q_{2}}}{\frac{1}{2}{\left({µ}_{p}C_{ox}\frac{W}{L}\right)}_{Q1}{\left(V_{{GS}_{1}}-V_{TH}\right)}^{2}{\left(1+lV_{ds}\right)}_{Q_{3}}}\approx \;\frac{{\left(\frac{W}{L}\right)}_{Q_{2}}}{{\left(\frac{W}{L}\right)}_{Q_{1}}}$$

being *V_GS_*_1_ = *V_GS_*_2_.

**Figure 4:**
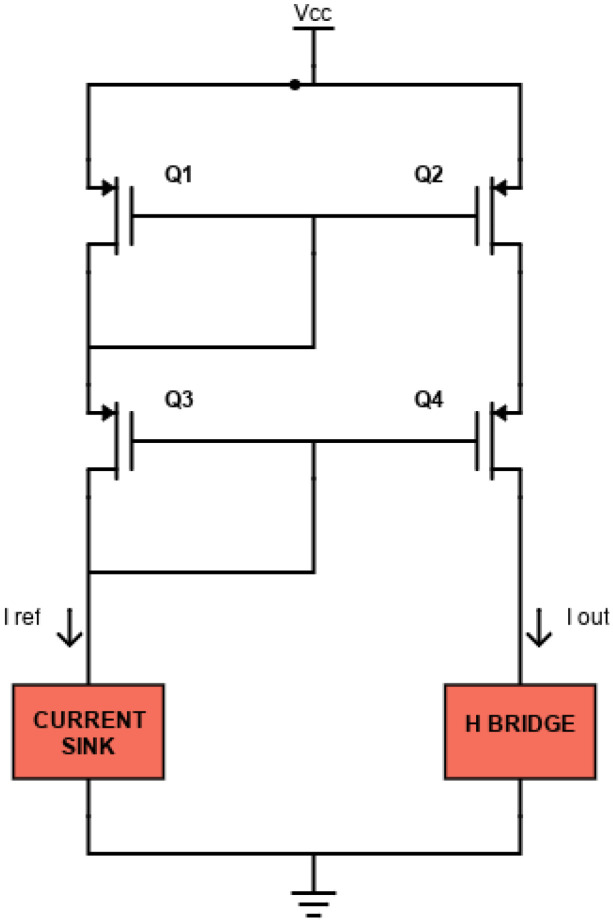
Schematic of the cascode current mirror circuit.

Using two MOSFETs with the same aspect ratio *W*/*L*, *I_OUT_* is almost equal to *I_REF_* (from Equation (5)). For even more accurate applications and especially to avoid the temperature dependence, a cascode current mirror can be used, as shown in Figure 4. In this configuration, transistors also operate in saturation. The cascode mirror ensures an improved output resistance and better accuracy because transistors *Q*_3_ and *Q*_4_ stabilize the drain-source voltages of the mirroring transistors (*Q*_1_ and *Q*_2_) by buffering them from variations in the output voltage, thus ensuring a more accurate current mirroring operation.

**Figure 5:**
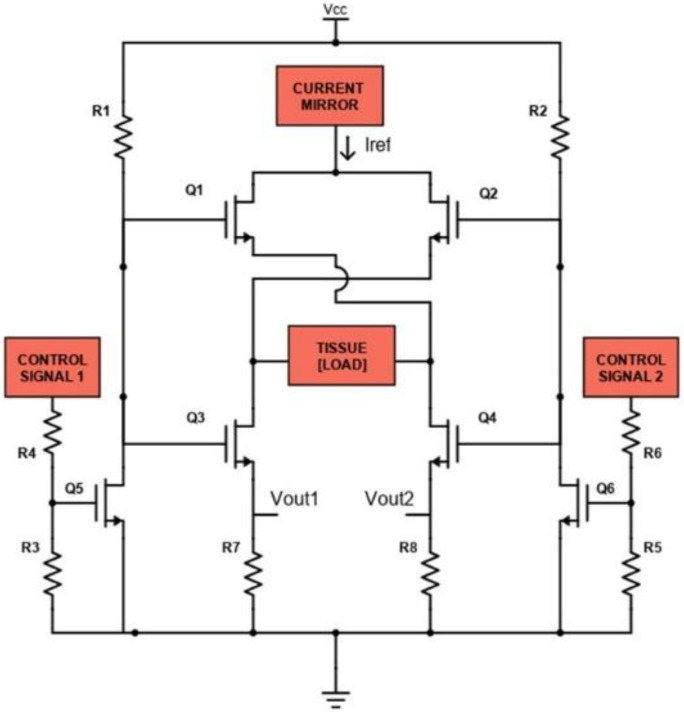
Schematic of the H-bridge circuit.

The H-bridge block is realized as shown in Figure 5.

The H-bridge consists of six controlled switches represented by the MOSFETs *Q*_1_, *Q*_2_, *Q*_3_, *Q*_4_, *Q*_5_ and *Q*_6_ (N-MOS: FDN86246, Onsemi, Scottsdale, AZ, USA). The function of these MOSFETs is to control the flow of current *I_REF_* through the load, represented by human tissue. When MOSFETs *Q*_1_ and *Q*_3_ are ON (and *Q*_2_ and *Q*_4_ OFF), a positive voltage is applied across the load, so *I_REF_* will flow from the left to the right (according to Figure 5), through the tissue; when MOSFETs *Q*_2_ and *Q*_4_ are ON (and *Q*_1_ and *Q*_3_ OFF), the applied voltage is reversed and *I_REF_* will flow on the left branch, through the tissue. *Q*_1_ and *Q*_3_ are simultaneously controlled by the N-MOS *Q*_5_, while *Q*_2_ and *Q*_4_ are simultaneously controlled by the N-MOS *Q*_6_. Control signals applied on *Q*_5_ and *Q*_6_ are based on square waves with a specific duty cycle, frequency and relative phase shift, generated by a µC (SAM3X8E ARM Cortex-M3, Atmel Corporation, San Jose, CA, USA) with 3.3 V logic levels. The shunt resistances *R*_7_ and *R*_8_ are used to measure the biphasic current flowing thorough the tissue. Voltages *V_OUT_*_1_ and *V_OUT_*_2_ are applied to two INA (INA819, Texas Instruments, Dallas, TX, USA), whose amplification has been set to 30 dB, to be amplified and acquired by the µC.

The desired current *I_REF_* is set applying a specific *V_in_* to the voltage controlled current sink block, through the internal DAC of the µC. The produced *I_REF_* will be mirrored by the current mirror stage to the H-bridge. Controlling the H-bridge with specific square wave signals makes it possible to inject *I_REF_* both in the monophasic and in the biphasic mode.

A PCB was designed using the EDA software Autodesk Eagle (Version 9.6.2, Autodesk Inc., San Francisco, CA, USA) and fabricated to test the performances of the developed electronic interface. During the test procedures, control signals were generated by the µC while measurements of the output signals were visualized using an oscilloscope (HDO6054-MS, Teledyne LeCroy, New York, NY, USA).

The evaluation of the performances of the developed interface was performed using a known load impedance Z; the testing impedance consists of a resistance of 1 kΩ. To evaluate the flexibility of the system, the test load was stimulated with both monophasic and biphasic current pulse bursts with a symmetrical and asymmetrical shape.

The tested stimulation patterns have the following characteristics: a burst of 10 monophasic current pulses with a frequency of 1 kHz and an amplitude of 1 mA (each current path of the h-bridge was tested) and a duty cycle of 25, 50 and 75%; a burst of 10 biphasic symmetrical current pulses with a frequency of 1 kHz and an amplitude of 1 mA; a burst of 10 biphasic asymmetrical current pulses with a frequency of 1 kHz, an amplitude of 1 mA and a relative duty cycle with a ratio equal to 1:3 and 3:1. Moreover, in order to verify that the injected current was equal to the desired current *I_REF_*, the test impedance was stimulated with bursts of 10 monophasic current pulses with an amplitude ranging from 0.1 to 10 mA (0.1, 0.5, 1, 2, 3, 4, 5, 6, 7, 8, 9, 10 mA) and a frequency of 1 kHz and a duty cycle of 50%. The amplitude range was chosen according to the typical range, between 0.1 and 4 mA, reported in the literature for reverse iontophoresis applications [17].

**Figure 6:**
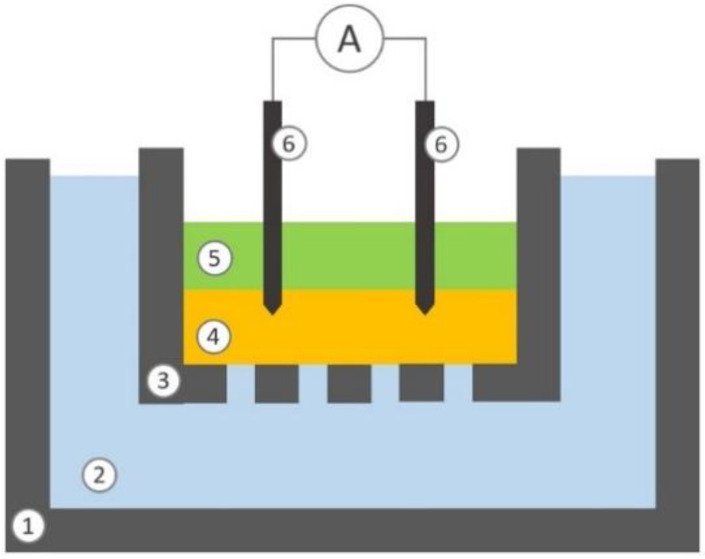
Schematic representation of the section view of the experimental setup: lower chamber (1), liquid phase contained in the lower chamber (2), upper chamber (3), hydrogel septum (4), liquid phase contained into the upper chamber (5), two electrodes (6).

Moreover, to test the system in a simulated environment, electrical stimulation was used to boost a mimicked physiological diffusion process. A custom structure, such as a reverse Franz diffusion cell, was built up, as shown in Figure 6.

The lower donor chamber (10 mL), made of polystyrene, is filled with TrisHCl buffer at pH 7.4 containing 140 mM of NaCl, 4 mM KCl and 20 mM of L-ascorbic acid (Merck KGaA, Darmstadt, Germany). The upper receptor chamber (1 mL), made of polylactide (PLA), is filled with TrisHCl buffer at pH 7.4, containing only NaCl and KCl; the bottom of the upper chamber is perforate to allow passive diffusion. The two chambers are separated by a layer of hydrogel, made of agarose 0.8% (*w*/*v*) dissolved in TrisHCl buffer at pH 7.4 containing 140 mM of NaCl and 4 mM KCl, with a thickness of 3 mm. The lower chamber simulates peripheral vessels and the hydrogel contained in the upper cell simulates the dermis. The buffer composition is thus defined to reflect a simplified model of blood and ISF systems. Two stainless steel needles are used as electrodes. The electrode tips are inserted 1 mm into the hydrogel upper layer.

Current stimulations of 500 and 1000 µA are applied for 30 min to the dermal simulated environment, independently. To compare the electro-stimulated diffusion against the spontaneous process, the passive diffusion phenomenon is allowed to occur for the same amount of time (30 min), where no stimulation is applied. At the end of the experiment the concentration of ascorbic acid into the liquid phase of the upper chamber is measured using a specific spectrophotometric assay [35].

The stimulation parameters were selected by analysing the most recent literature. The works taken into consideration are shown in the Table 1.

## 3. Results

### 3.1. Monophasic Test

**Figure 7:**
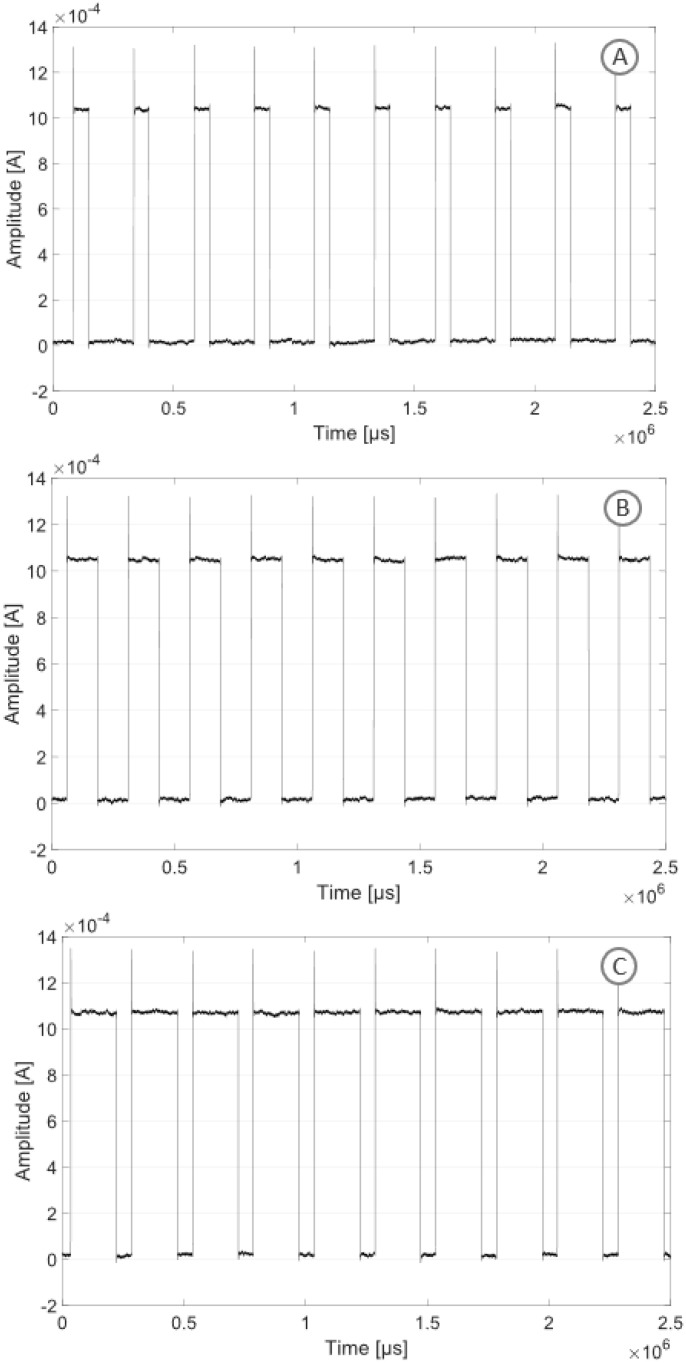
Monophasic stimulation pattern with three different duty cycles and an amplitude of 1 mA: duty cycle of 25% (**A**), duty cycle of 50% (**B**), duty cycle of 75% (**C**).

Figure 7 shows the stimulation pattern obtained in monophasic mode with a duty cycle of 25, 50 and 75%, respectively. A current square wave with a frequency of 1 kHz, a duty cycle of 25, 50 and 75%, respectively, and an amplitude of 1 mA was recorded.

### 3.2. Biphasic Test

**Figure 8:**
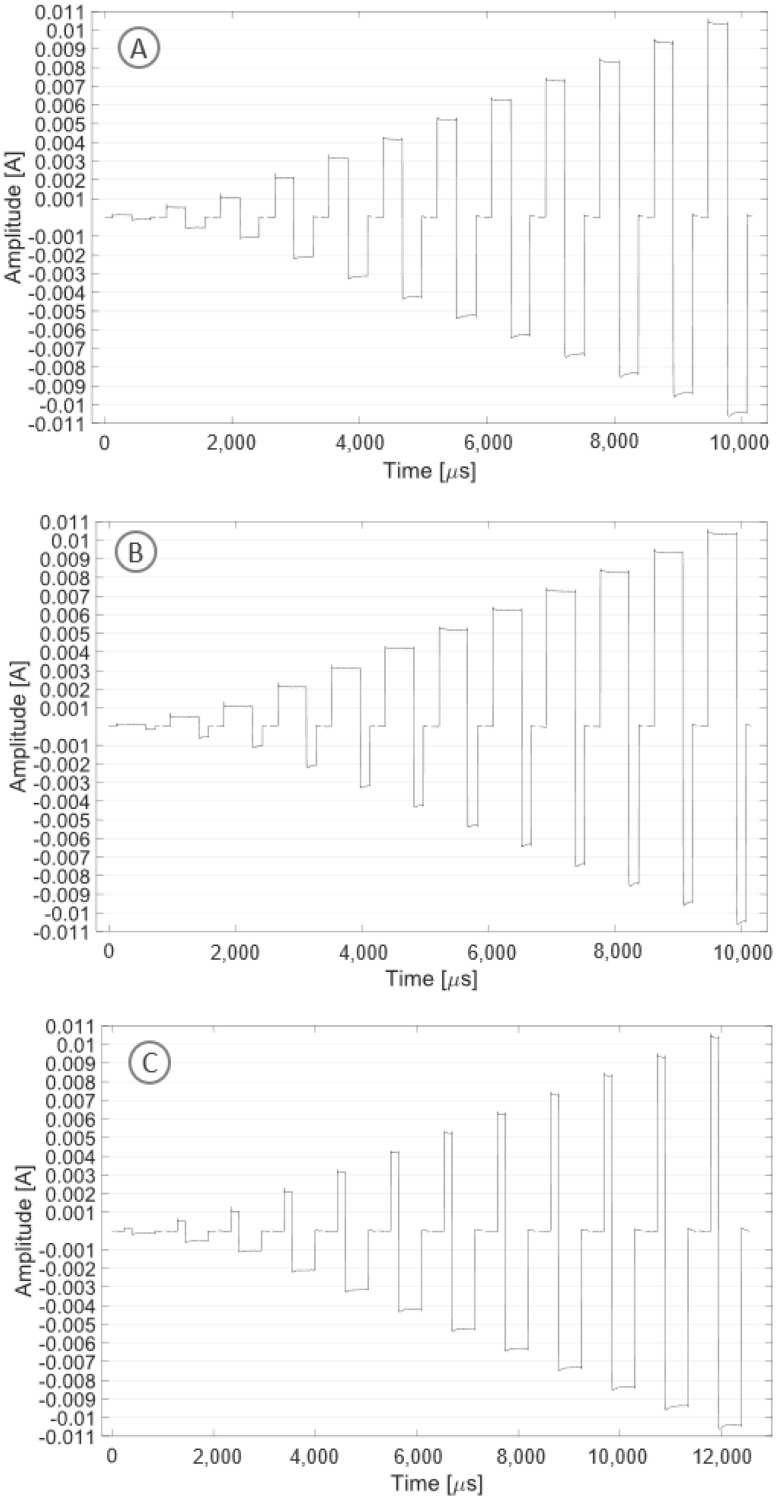
The plot shows one period for each tested stimulation pattern with increasing current amplitude (0.1, 0.5, 1, 2, 3, 4, 5, 6, 7, 8, 9, 10 mA): symmetrical pattern (**A**), asymmetrical pattern with a ratio of 3:1 (**B**), asymmetrical pattern with a ratio of 1:3 (**C**).

Figure 8A shows the stimulation pattern obtained in biphasic mode with symmetrical current pulses. A current square wave with both symmetrical positive and negative amplitudes with a modulus ranging from 0.1 to 10 mA and a frequency of 1 kHz was recorded. Figure 8B shows the stimulation pattern obtained in biphasic mode with asymmetrical current pulses. A current square wave with asymmetrical timing (with a ratio of 3:1), amplitudes with a modulus ranging from 0.1 to 10 mA and a frequency of 1 kHz was recorded. Figure 8C shows the stimulation pattern obtained in biphasic mode with asymmetrical current pulses. A current square wave with asymmetrical timing (with a ratio of 1:3), amplitudes with a modulus ranging from 0.1 to 10 mA and a frequency of 1 kHz was recorded.

### 3.3. Injected Currents

**Figure 9:**
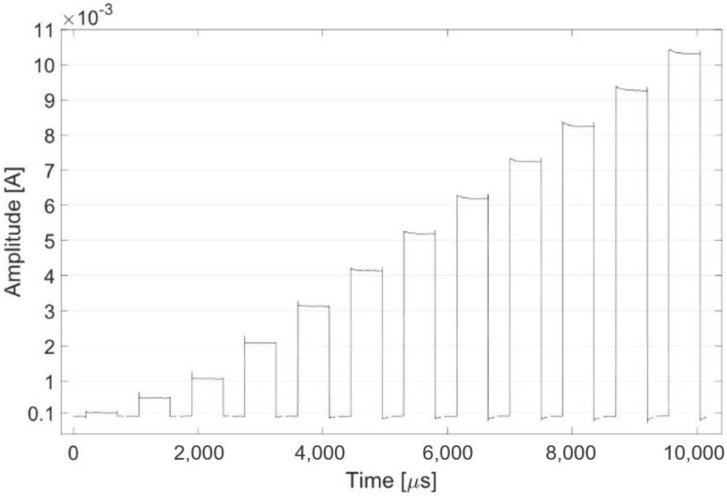
The plot shows one period for each tested stimulation pattern with increasing current amplitude (0.1, 0.5, 1, 2, 3, 4, 5, 6, 7, 8, 9, 10 mA).

Figure 9 shows the stimulation pattern obtained in monophasic mode with a duty cycle of 50%. A current square wave with a frequency of 1 kHz, a duty cycle of 50% and an amplitude ranging from 0.1 to 10 mA was recorded.

**Figure 10:**
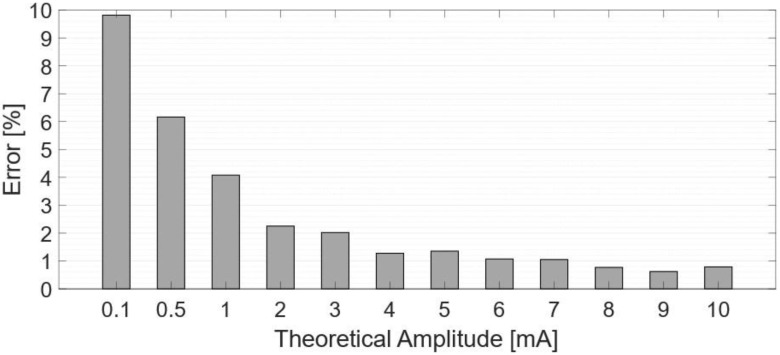
Percentage error between measured and theorical values.

Figure 10 shows the % relative error between the desired currents and the measured injected currents.

### 3.4. Reverse Iontophoresis Scenario

**Figure 11:**
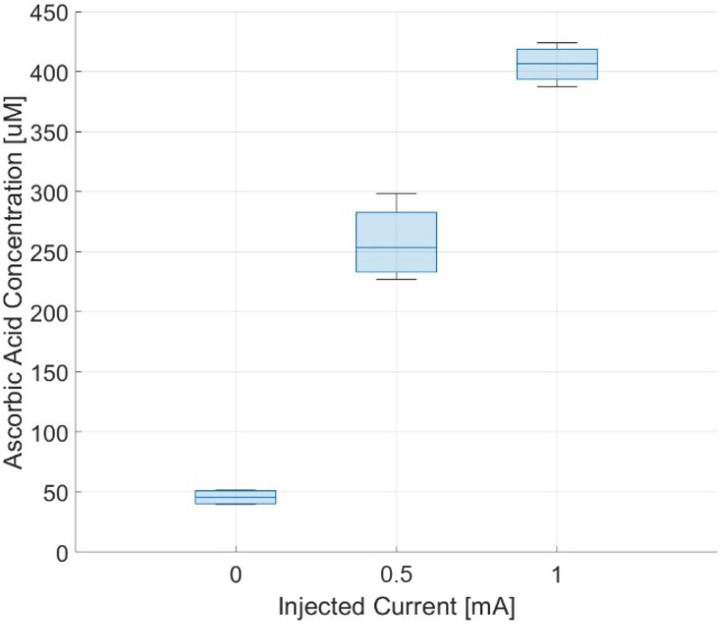
Measured concentrations of ascorbic acid in the receptor chamber after 30 min.

Figure 11 shows the concentrations of ascorbic acid measured in the upper chamber at the end of the experiment. The concentrations obtained with the stimulation protocol are significantly higher (from 5 to 8 times) than that obtained with the spontaneous process and someway proportional to the intensity of the applied currents.

## 4. Discussion and Conclusions

In this paper, we have described the development and realization of a high-voltage current stimulator suitable for reverse iontophoresis applications. Different types of current stimuli can be applied; the intensity, the frequency and the duty-cycle of the stimulation patterns can be programmed. Both monophasic and biphasic pulses can be used with custom duty cycles and amplitudes, ranging from 0.1 to 10 mA (fully compatible with current ranges found in literature), with a maximum error of about 10% at the minimum intensity. The intensity of the injected current can be measured in real-time to control the system. The developed device has a theoretical voltage compliance of about ±120 V and is capable of driving a maximum load of about 1 MΩ at 0.1 mA and of about 10 kΩ at 10 mA.

Several studies found in the literature were taken into consideration to evaluate and compare the performances of the electronic interface developed in this study. Considering the research reported in Table 2, the developed electronic interface features the following characteristics: a wide range of stimulation frequencies with good voltage compliance, a low circuital complexity because of its single supply, high voltage op-amps or transformers and excellent safety features.

The electronic interface was specifically designed to avoid electrical injuries; in fact, when no current is injected, the load is floating, so is not connected either to the 120 V or to the ground, avoiding the possibility of unwanted shorts.

Moreover, the applicability of the developed electronic interface was tested in a simulated environment demonstrating that the device can extract analytes from peripheral vessels to dermis, exploiting the reverse iontophoresis effect. In this case, the effect of reverse iontophoresis would be twofold. On the one hand, the application of the current stimulus allows the analyte concentration to increase within the ISF, facilitating its detection, and on the other hand, it allows the time lag to decrease between the analyte concentration present in the blood compared to that present in the ISF (for example, in the case of glucose there is a time lag of 15/20 min [42]), allowing for measurements that are closer to reality. It is important to note that in vivo tests on biological tissues or animal models would further validate the effectiveness and safety of the developed stimulator, but unfortunately, these kinds of experiments are difficult to conduct at this stage of development; we mainly tried to verify the effectiveness of the developed electronic interface in a controlled environment to lay the foundation for further developments. Moreover, testing a broader range of analytes could confirm the applicability of the developed device in a broader range of applications.

In this work, a constant current was used to extract analytes. It would be very interesting to test different and more complex stimulation patterns in order to evaluate their effect on the extraction performance. In addition, it would also be important to optimize the stimulation patterns with a view to minimizing skin irritation, which is one of the major obstacles to the real application of reverse iontophoresis for continuous monitoring. There may be different ways to reduce the skin irritation, as reported in Zheng et al. [27]: use of more suitable hydrogels, improvement of skin permeability and use of appropriate current stimuli. Regarding this last factor, applying too much current for an extended period into the skin will cause irritation, erythema, etc. According to Giri et al. [32], the current intensity applied to the skin should range from 0.1 to 0.3 mA. Moreover, according to Li et al. [43], continuous direct current applied to the skin can also cause irritation. To avoid this problem, pulsed direct current can be used for reverse iontophoresis. By controlling the time of each pulse and the time of rest, a certain recovery time can be given to the skin. For these reasons, we chose to make a reverse iontophoresis stimulator that was capable of applying current stimuli so that we could have total control over the amount of current injected. Another concept to be considered concerns the charge balance within the stimulated tissues. As reported in Xu et al. [44], the skin irritation in iontophoresis is partly due to the polarization caused by continuous DC induction. By applying current in a high-frequency pulse waveform, especially by briefly reversing the current during each cycle, this polarization can be avoided; thus, in order to avoid skin irritation and prevent damages, the charge balance must always be zero. This can be achieved by using biphasic stimulation currents that inject equal amounts of charge in alternating directions. For this reason, we decided to develop a stimulator that was capable of applying biphasic current stimulation. Obviously, further studies on these specific aspects are needed to optimize the stimulation parameters and minimize irritation.

Further developments are necessary to embed the proposed electronic interface into a wearable system. At this stage, the developed interface is designed to be implemented with discrete components, and it is not small enough to be embedded into a wearable device. Further steps would be needed to integrate all the electronic circuits into an integrated chip, which is completely feasible: the electronic interface is mainly designed and developed with MOSFETs and resistors that can be easily integrated into a chip. The only component that is difficult to miniaturize is the high voltage power supply, as it is based on the commercial power supply module R05-100B (RECOM Power GmbH, Gmunden, Austria), the size of which is 30 × 20 × 9 mm. It is also important to emphasize that this component offers performance that greatly satisfies the voltage compliance required for reverse iontophoresis applications using micro-needles, so assuming future miniaturization of the system, the current voltage supply could be replaced with a custom circuit designed with miniaturization as its primary objective, also against voltage compliance.

## Figures and Tables

**Table 1:** Typical reverse iontophoresis parameters found in literature.

Target	Current Stimulus	Extraction Time	Electrode Material	Electrode Type	Ref
Ascorbic Acid	0.5/1 mA	30 min	Stainless Steel	Needles	This Work
Glucose	1 mA	9 min	Ag/AgCl	Microneedles	[28]
Glucose	0.5 mA	5 min	Ag/AgCl	Microneedles	[30]
Glucose	0.01 mA	4 min	Ag/AgCl	Flat Electrode	[31]
Levetiracetam	0.9 mA	1 h	Ag/AgCl	Cylindrical Electrode	[32]
Cortisol	0.1 mA/cm^2^	3 h	Ag/AgCl	Bare Wire	[34]

**Table 2:** Comparison chart of electronic interfaces for electrical stimulation found in literature.

Reference	Frequency Range	Current Range	Circuit Highlights	Safety Features
This work	Up to 50 kHz	±120 V	VCCS Current mirror H-bridge Single power supply	Load grounded when no stimulation is performed Continuous monitoring of injected current
Cheng et al. [36]	200 Hz	±200 V	TIMER 555 (2) Op-amp (4) Transformer (1) Single power supply	Continuous feedback
Velloso and Souza [37]	20–200 Hz	±160 V	Op-amp Darlington BJT Transformer Dual power supply	Continuous feedback
Qu et al. [38]	Up to 50 kHz	±100 V	VCCS H-bridge Single power supply	Continuous feedback
Masdar et al. [39]	Up to 50 kHz	±120 V	High voltage op-amp Step-up transformer Dual power supply	Load grounded when no stimulation is performed Continuous monitoring of injected current
Brunetti et al. [40]	Up to 100 Hz	±250 V	High voltage op-amp Dual power supply	Load grounded when no stimulation is performed
Karpul et al. [41]	Up to 1 kHz	±60 V	High voltage op-amp Dual power supply	Continuous feedback

## Data Availability

Data are available upon reasonable request.
